# Evaluation of antihypertensive medications use and survival in patients with ovarian cancer: a population-based retrospective cohort study

**DOI:** 10.1186/s12905-024-02983-7

**Published:** 2024-03-04

**Authors:** Rūta Everatt, Irena Kuzmickienė, Birutė Brasiūnienė, Ieva Vincerževskienė, Birutė Intaitė, Saulius Cicėnas, Ingrida Lisauskienė

**Affiliations:** 1 Laboratory of Cancer Epidemiology, National Cancer Institute, Baublio 3B, Vilnius, LT-08406 Lithuania; 2 Department of Medical Oncology, National Cancer Institute, Vilnius, Lithuania; 3 Faculty of Medicine, Vilnius University, Vilnius, Lithuania; 4 Cancer Registry, National Cancer Institute, Vilnius, Lithuania; 5 Department of Gynaecologic Oncology, National Cancer Institute, Vilnius, Lithuania; 6 Department of Thoracic Surgery and Oncology, National Cancer Institute, Vilnius, Lithuania; 7 Institute of Clinical Medicine, Faculty of Medicine, Vilnius University, Vilnius, Lithuania

**Keywords:** Ovarian cancer survival, Antihypertensive drugs, Cohort study, Lithuania

## Abstract

**Background:**

Despite declining mortality in most countries and in Lithuania, ovarian cancer burden has remained high. Studies have indicated that antihypertensive medications use may help to improve ovarian cancer survival, however findings remain controversial. The aim of the study was to analyse the association between post-diagnosis antihypertensive medications intake and cancer-specific survival in ovarian cancer patients.

**Methods:**

This retrospective cohort study included 588 ovarian cancer cases diagnosed between 2013 and 2015. Hazard ratios (HR) and corresponding 95% confidence intervals (95%CI) were estimated using multivariable Cox proportional hazards models to assess associations between antihypertensive medications and ovarian cancer-specific mortality.

**Results:**

In total, 279 (47%) patients died during the follow-up; 242 (87%) of them died due to ovarian cancer. The risk of ovarian cancer death was reduced in angiotensin-converting enzyme inhibitors (ACE inhibitors) users vs. non-users (HR 0.55, 95% CI: 0.36–0.83). Subgroup analysis showed better ovarian cancer survival in higher dose ACE inhibitors users (HR 0.46, 95% CI: 0.28–0.77, p for trend 0.002); the effect was also stronger in age 51–65 years, stage I–III, surgery or chemotherapy treatment, pre-diagnosis ACE inhibitor users’ and pre-diagnosis hypertension subgroups. The risk of cancer-specific death was slightly lower among calcium-channel blocker and angiotensin-receptor blocker users and higher among beta-blocker users as compared to non-users, however chance and confounding could not be ruled out. We found no association between the use of centrally and peripherally acting antiadrenergic agents and diuretics and risk of ovarian cancer-specific mortality.

**Conclusions:**

Our findings imply that post-diagnosis use of ACE inhibitors may be associated with reduced ovarian cancer-specific mortality; however, further research is needed for the comprehensive assessment.

**Supplementary Information:**

The online version contains supplementary material available at 10.1186/s12905-024-02983-7.

## Background

In 2020, 313,959 new ovarian cancer cases and 207,252 deaths from ovarian cancer were recorded worldwide [1]. Ovarian cancer represents 3.4% of the global cancer burden in women. In Lithuania, ovarian cancer was the 7th most common cancer (352 new cases, 4.2%) and 5th most common cause of cancer death (257 deaths, 7%) in 2020 [1]. With an age-standardized incidence rate of 11.8 per 100,000 and mortality rate 7.2 per 100,000, Lithuania ranked 8th for the highest ovarian cancer incidence and 6th for the highest mortality in the world in 2020 [1]. Ovarian cancer mortality rates have been declining in most countries and in Lithuania, suggesting reduction in the prevalence of risk factors or improvements in diagnosis and treatment [1, 2]. Being overweight or obese, not bearing children, early menarche (before the age of 12), late natural menopause (after the age of 55), hormone therapy, smoking and family history have previously been associated with an increased risk of ovarian cancer [3–5]. There is also some evidence that breastfeeding might decrease the risk of ovarian cancer [3].

Ovarian cancer is associated with poor prognosis; therefore, research is needed to find novel or existing medications that may help to improve survival. Hypertension is a common comorbidity in patients with cancer. A number of large cohort studies on the association between the antihypertensive medication use and ovarian cancer death risk showed a significant beneficial effect of post-diagnostic angiotensin-converting enzyme inhibitors (ACE inhibitors) use on cancer-specific survival compared to not users [6, 7]. Moreover, in a study of women 66 + years of age with ovarian cancer, lower cancer-specific mortality was shown in patients who received ACE inhibitors or diuretics during the year following a cancer diagnosis [8]. A protective effect of angiotensin-receptor blockers (ARBs) has been observed among epithelial ovarian cancer patients by Cho et al. [9]; however, no clear associations between post-diagnostic ARBs or beta-blockers (BBs) use and ovarian cancer mortality were found in cohort studies by Santala et al. or Huang et al. [6, 7]. The results of several recent studies suggested survival benefits for BBs users, particularly for nonselective BBs users [8, 10, 11]. In contrast, some investigators found no association [12] or a significant positive association, especially for selective BBs [10, 13–15]. In recent meta-analyses it was concluded, that there is insufficient evidence regarding the association between ACE inhibitors or BBs and ovarian cancer survival [12, 16]. Consumption of other antihypertensive drug groups (calcium channel blockers (CCBs) or diuretics) was not associated with a decreased ovarian cancer death risk, except the elevated risk observed with furosemide use [6, 7, 9]. We found no studies aiming to examine the role of centrally and peripherally acting antiadrenergic agents (SNS-AH) in ovarian cancer survival.

There is significant heterogeneity in the results from epidemiological studies and the evidence on the impact of age, stage or follow-up time length is sparse. Furthermore, studies in populations with widespread SNS-AH use or selective BBs use as a standard practice for arterial hypertension treatment are lacking. We therefore investigated the association between post-diagnostic antihypertensive medications intake and ovarian cancer-specific survival.

## Methods

### Study subjects

In this retrospective cohort study we included ovarian cancer cases diagnosed between 2013 and 2015, identified from Lithuanian Cancer Registry (NCR). This Registry contains information on date and methods of diagnosis, tumour characteristics, date and cause of death. For the present study, ovarian cancer code C56 was used (International Statistical Classification of Diseases,10th Revision, ICD-10). In all, 1184 records were available for analysis. Cases were subjects with primary, first-incident, histologically confirmed ovarian cancer. Individuals with multiple cancers, age less than 25 years or more than 80 years, death before start of follow-up, were excluded. We also excluded cases with diagnosis based on death certificate. The final number of participants, included in the current analysis, was 588.

Information on demographic factors (age at the time of diagnosis, location of residence), cancer-related factors (including tumor histology, stage at diagnosis), and other potential confounders was available from NCR. All eligible cases were classed as serous adenocarcinoma (using ICD for Oncology (ICD-O) morphology codes 84413, 84503, 84603 and 84613), mucinous adenocarcinoma (ICD-O 84703, 84803, 84813 and 84903), endometrioid adenocarcinoma (83803; 85703) or Other (81403, 83103, 82603, 84403, etc.). In addition, information on receipt of cancer treatment including surgery, chemotherapy and radiotherapy within 1 year after cancer diagnosis as well as on other health-related factors was collected from NHIF.

To estimate the Charlson Comorbidity Index (CCI), comorbidities were identified from the NHIF database, including myocardial infarction, congestive heart disease, peripheral vascular disease, cerebrovascular disease, chronic pulmonary disease, rheumatological disease, dementia, hemiplegia, diabetes, diabetes with complications, renal disease, mild liver disease, moderate or severe liver disease, peptic ulcer disease, and AIDS [17]. The CCI was calculated based on the information during the 1 year prior to diagnosis period. In addition, statin, antidiabetic and antithrombotic medicines use (dates of prescription and purchase, dose, strength and amount of the drug) were also identified from NHIF database as previous studies have suggested these drugs could reduce mortality in patients with ovarian cancer [15, 18, 19].

### Antihypertensive medication use

Data on patients’ use of antihypertensive medications (SNS-AH, diuretics, BBs, CCBs, ARBs and ACE inhibitors) during 2012–2016 was obtained by linkage with the National Health Insurance Fund (NHIF) database. The NHIF’s database contains information on all purchases in outpatient settings of physician-prescribed reimbursed medicines, and covers up to 100% of insured Lithuanian population (about 98% of population is covered by health insurance) [20]. The following information about each purchase was obtained: drug’s Anatomical Therapeutic Chemical (ATC) classification system code, the brand name of the medicine, dates of prescription and purchase, also dose, strength and amount of the drug (pills, ampoules, inhalators and etc.). The daily defined doses (DDD) in each prescription were calculated by multiplying the quantity by the strength (in mg) and dividing by the mg in a DDD from the World Health Organization [21]. In order to account for dose, the total number of DDDs in each antihypertensive medication class for the first year after ovarian cancer diagnosis were calculated.

### Statistical analyses

The primary exposure of interest was use of antihypertensive medications, categorized into the following major classes: (a) C02, centrally and peripherally acting antiadrenergic agents (SNS-AH); (b) C03, diuretics; (c) C07, beta blocking agents (BBs); (d) C08, calcium channel blockers (CCBs), and (d) C09, agents acting on the renin–angiotensin system, i.e.: angiotensin converting enzyme inhibitors (ACE inhibitors) and angiotensin receptor blockers (ARB). When fixed-dose combinations (several drugs in the same tablet) were used (e.g. BB and thiazides), each combined agent was included into respective antihypertensive medications class. Participants were considered users of a given type of antihypertensive medication if they had record of one or more purchases for a drug in that antihypertensive medication class during the first year following cancer diagnosis. Non-users of the antihypertensive drug class being investigated were classified as reference group. Dose dependence was evaluated by stratifying users of each antihypertensive medication class into two groups (low and high usage), the cut-point used was the median DDD amount use in antihypertensive medications class reached during the first year after diagnosis. To avoid immortal time bias [22], we applied a one year fixed baseline period during which exposure was defined and after which person-time and events were counted.

The association between antihypertensive medication use within one year after diagnosis and risk of ovarian cancer-specific, overall and non-cancer death was estimated using hazard ratios (HRs) and 95% confidence intervals (CIs). The HRs were calculated using Cox proportional hazards regression models. The time scale was time beginning one year after ovarian cancer diagnosis. The exit time was the date at death, or the end of follow-up December 31st, 2020.

Results of analysis are presented with minimal adjustment or full adjustment. Fully adjusted models included mutual adjustment for SNS-AH, diuretics, BBs, CCBs, ARBs, ACE inhibitors. Other covariates included potential confounding: age at diagnosis (25–50 years, 51–55, 56–60, 61–65, 66–70, 71–75, 76–80 years), place of residence (urban, less urban, rural), CCI (0, 1, 2, 3+), stage at diagnosis (I, II, III, IV, unknown), histology (serous adenocarcinoma, mucinous adenocarcinoma, endometrioid adenocarcinoma or Other), receipt of surgery within a year after cancer diagnosis (yes/no), receipt of chemotherapy within a year after cancer diagnosis (yes/no), use of diabetes medications metformin and insulin in the year prior to diagnosis (yes/no), statins in the year prior to diagnosis (yes/no), anticoagulants in the year prior to diagnosis (yes/no), and antihypertensive medications in the year prior to diagnosis (yes/no). P-values for trend were calculated by adding the ordinal antihypertensive medications usage variable (low/high) as continuous into the regression analyses. We assessed the proportional hazards assumptions by inspecting the log(− log) survival curves and using the Schoenfeld test for the exposure and adjustment variables; no violation of proportional hazards was observed.

Lag-time analysis was performed by excluding cases within the first 2 years of the follow-up period. In addition, stratified analyses were conducted among subgroups by age at diagnosis (51–65 years and > 65 years), stage at diagnosis (I-III and IV), histological type (serous, other), cancer treatment (surgery, chemotherapy) and other covariates. We also conducted sensitivity analyses restricted to women over 50 years old at diagnosis, as the prevalence of hypertension and use of antihypertensives is increased in older adults.

All analyses were performed using STATA/IC, 11.0 by STATA software (Stata Corporation, College Station, Texas, USA). All statistical tests were based on 2-sided probability, and, if less than 0.05, considered statistically significant.

## Results

### Patient characteristics

A total of 588 ovarian cancer patients were included in analyses. Baseline characteristics of the participants according to post-diagnosis antihypertensive medications consumption are summarized in Table 1. Overall, as compared with non-users, users were more likely to be older at the time of diagnosis, have more comorbidities and use statins, anticoagulants and antidiabetic medications.

**Table 1:** Participant characteristics

	All patients	SNS-AH		Diuretics		Beta blockers		Calcium channel blockers		Angiotensin receptor blockers		ACE inhibitors	
		Yes	No	Yes	No	Yes	No	Yes	No	Yes	No	Yes	No
**Number of women, n (%)**	588	51 (8.7)	537 (91)	117 (20)	471 (80)	166 (28)	422 (72)	73 (12)	515 (88)	62 (10)	526 (89)	145 (25)	443 (75)
**Age at diagnosis, mean**	57.5	67.3	56.6	65.5	55.5	62.8	55.4	65.0	56.5	64.6	56.7	64.1	55.3
**Urban/rural, n (%)**													
Urban	286 (49)	29 (57)	257 (48)	46 (39)	240 (51)	76 (46)	210 (50)	36 (49)	250 (48)	28 (45)	258 (49)	65 (45)	221 (50)
Less urban	143 (24)	11 (22)	132 (25)	31 (26)	112 (24)	38 (23)	105 (25)	17 (23)	126 (24)	14 (23)	129 (24)	34 (23)	109 (25)
Rural	159 (27)	11 (22)	148 (27)	40 (34)	119 (25)	52 (31)	107 (25)	20 (27)	139 (27)	20 (32)	139 (26)	46 (32)	113 (25)
**Tumor histology, n (%)**													
Serous	365 (62)	35 (69)	330 (61)	80 (68)	285 (60)	120 (72)	245 (58)	52 (71)	313 (61)	42 (68)	323 (61)	102 (70)	263 (59)
Mucinous	23 (4)	1 (2.0)	22 (4.1)	3 (2.6)	20 (4.2)	6 (3.6)	17 (4.0)	3 (4.1)	20 (3.9)	2 (3.2)	21 (4.0)	4 (2.8)	19 (4.3)
Endometrioid	58 (10)	1 (2.0)	57 (11)	5 (4.3)	53 (11)	8 (4.8)	50 (12)	3 (4.1)	55 (11)	-	58 (11)	11 (7.6)	47 (11)
Other	142 (24)	14 (27)	128 (24)	29 (25)	113 (24)	32 (19)	110 (26)	15 (20)	127 (25)	18 (29)	124 (24)	28 (19)	114 (26)
**Stage at diagnosis, n (%)**													
I	143 (24)	13 (25)	130 (24)	25 (21)	118 (25)	37 (22)	106 (25)	19 (26)	124 (24)	18 (29)	125 (24)	32 (22)	111 (25)
II	48 (8.2)	2 (3.9)	46 (8.6)	6 (5.1)	42 (8.9)	8 (4.8)	40 (9.5)	5 (6.8)	43 (8.3)	7 (11)	41 (7.8)	7 (4.8)	41 (9.3)
III	279 (47)	24 (47)	255 (47)	62 (53)	217 (46)	93 (56)	186 (44)	33 (45)	246 (48)	28 (45)	251 (48)	73 (50)	206 (46)
IV	98 (17)	11 (22)	87 (16)	17 (14)	81 (17)	21 (13)	77 (18)	13 (18)	85 (16)	8 (13)	90 (17)	26 (18)	72 (16)
Unknown	20 (3.4)	1 (2.0)	19 (3.5)	7 (6.0)	13 {2.8}	7 (4.2)	13 (3.1)	3 (4.1)	17 (3.3)	1 (1.6)	19 (3.6)	7 (4.8)	13 (2.9)
**Surgery, n (%)**	498 (85)	40 (78)	458 (85)	95 (81)	403 (85)	132 (79)	366 (87)	64 (88)	434 (84)	51 (82)	447 (85)	127 (88)	371 (84)
**Chemotherapy, n (%)**	516 (88)	45 (88)	471 (88)	100 (85)	416 (88)	149 (90)	367 (87)	58 (79)	458 (89)	52 (84)	464 (88)	127 (88)	389 (88)
**Comorbidities** ^**a**^													
Hypertension, n (%)	248 (42)	47 (92)	201 (37)	98 (84)	150 (32)	138 (83)	110 (26)	63 (86)	185 (36)	57 (92)	191 (36)	122 (84)	126 (28)
CVD^b^, n (%)	294 (50)	47 (92)	247 (46)	103 (88)	191 (40)	143 (86)	151 (36)	64 (88)	230 (45)	58 (93)	236 (45)	125 (86)	169 (38)
Diabetes, n (%)	36 (6.1)	7 (14)	29 (5.4)	13 (11)	23 (4.9)	18 (11)	18 (4.3)	12 (16)	24 (4.7)	7 (11)	29 (5.5)	11 (7.6)	25 (5.6)
**CCI** ^**c**^, **n (%)**													
0	395 (67)	15 (29)	380 (71)	56 (48)	339 (72)	88 (53)	307 (73)	31 (42)	364 (71)	25 (40)	370 (70)	76 (52)	319 (72)
1	54 (9.2)	7 (14)	47 (8.7)	11 (9.4)	43 (9.1)	13 (7.8)	41 (9.7)	10 (14)	44 (8.5)	5 (8.1)	49 (9.3)	16 (11)	38 (8.6)
2	71 (12)	21 (41)	50 (9.3)	34 (29)	37 (7.9)	41 (24.7)	30 (7.1)	17 (23)	54 (10)	21 (34)	50 (9.5)	32 (22)	39 (8.8)
3+	68 (12)	8 (16)	60 (11)	16 (14)	52 (11)	24 (14)	44 (10)	15 (20)	53 (10)	11 (18)	57 (11)	21 (14)	47 (11)
**Use of other medications** ^**a**^													
Statins, n (%)	19 (3.2)	8 (15.7)	11 (2.0)	8 (6.8)	11 (2.3)	14 (8.4)	5 (1.2)	1 (1.4)	18 (3.5)	6 (9.7)	13 (2.5)	10 (6.9)	9 (2.0)
Metformin, n (%)	27 (4.6)	6 (11.8)	21 (3.9)	14 (12)	13 (2.8)	17 (10)	10 (2.4)	8 (11)	19 (3.7)	7 (11)	20 (3.8)	10 (6.9)	17 (3.8)
Insulin, n (%)	5 (0.8)	2 (3.9)	3 (0.6)	-	5 (1.1)	2 (1.2)	3 (0.7)	2 (2.7)	3 (0.6)	-	5 (0.9)	1 (0.7)	4 (0.9)
Anticoagulants, n (%)	13 (2.2)	5 (9.8)	8 (1.5)	8 (6.8)	5 (1.1)	8 (4.8)	5 (1.2)	4 (5.5)	9 (1.8)	3 (4.8)	10 (1.9)	8 (5.5)	5 (1.1)

^a^in the year prior to diagnosis

^b^Cardiovascular disease

^c^Charlson Comorbidity Index

Among participants, in 1-year post-diagnosis period 51 (8.7%) of patients were SNS-AH users, 117 (20%) - diuretics users, 166 (28%) - BBs users (all – selective BBs), 73 (12%) - CCBs users, 62 (10%) - ARBs users, and 145 (25%) were ACE inhibitors users (Table 1). Overall, 43.7% of participants used any type of antihypertensive medications within one year after diagnosis. Mean age at diagnosis was 57.5 years for all patients, there were significant differences between users and non-users. The mean age at diagnosis for SNS-AH, diuretics, BBs, CCBs, ARBs, and ACE inhibitors users was 67.3, 65.5, 62.8, 65.0, 64.6 and 64.1 years, respectively. However, further analysis showed significant mean age differences between users and non-users only among < 50 years old ovarian cancer patients, but not in older (51–65 or ≥ 65 years) age categories (data not shown). Serous adenocarcinoma was the main histologic type, detected for 365 (62%) patients, of which 50% was low grade serous carcinoma and 48% - serous carcinoma, NOS. There were no significant differences in histologic type between users and non-users of SNS-AH, diuretics, CCBs and ACE inhibitors, whereas serous adenocarcinoma was more frequent among BBs users (72% of users vs. 58% of non-users, *p* = 0.006) and ARB users (68% of users vs. 61% of non-users, *p* = 0.012). There was a balance in most of the antihypertensive medications user and non-user groups with regards to stage, majority of cases were stage III or IV.

### Survival

In total, 279 (47%) of patients died during the follow-up; 242 (87%) of them died due to ovarian cancer; the mean follow-up time was 3.9 years, maximum 7 years.

As shown in Table 2, the post-diagnostic use of SNS-AH and diuretics was not associated with the risk of cancer-specific mortality in patients with ovarian cancer (HR 1.05, 95% CI: 0.61–1.80; HR 1.03, 95% CI: 0.68–1.56, respectively). In BBs users, a borderline significantly increased risk was observed compared to BB non-users (HR 1.43, 95% CI: 0.98–2.08), the increase became more pronounced at low-dose use of BBs (HR 1.67, 95% CI: 1.10–2.53). Consumption of CCBs and ARBs was associated with not statistically significantly decreased ovarian cancer-specific death risk (HR 0.75, 95% CI: 0.45–1.23 and HR 0.73, 95% CI: 0.43–1.23, respectively), no dose-response relationship was observed. Post-diagnostic ACE-inhibitor users had a reduced ovarian cancer-specific death risk (HR 0.55, 95% CI: 0.36–0.83), the results also showed better ovarian cancer survival in higher dose users (HR 0.46, 95% CI: 0.28–0.77, p for trend 0.002). In addition, there was a decrease in all-cause mortality among users of ACE inhibitors compared with non-users, and no significant association between AH medication use and non-cancer mortality (Table 2).

**Table 2:** Antihypertensive (AH) medications use and risk of ovarian cancer, all-cause and non-cancer mortality

AH medica-tion use	Ovarian cancer mortality				All-cause mortality				Non-cancer mortality		
	Deaths/ Cases, n	HR (95% CI)			Deaths/ Cases, n	HR (95% CI)			Deaths/ Cases, n	HR (95% CI)	
		Age-adjusted	Fully adjusted^a^			Age-adjusted	Fully adjusted^a^			Age-adjusted	Fully adjusted^a^
**SNS-AH**											
Non-use	219/537	1.00	1.00		248/537	1.00	1.00		15/537	1.00	1.00
Use	23/51	0.96 (0.61; 1.50)	1.05 (0.61; 1.80)		31/51	1.08 (0.73; 1.59)	1.12 (0.71; 1.79)		5/51	1.97 (0.69; 5.67)	1.46 (0.41; 5.11)
low^b^	10/25	0.91 (0.48; 1.73)	1.01 (0.48; 2.10)		15/25	1.17 (0.68; 1.99)	1.19 (0.64; 2.19)				
high	13/26	1.00 (0.56; 1.79)	1.08 (0.56; 2.11)		16/26	1.00 (0.59; 1.69)	1.07 (0.59; 1.93)				
**Diuretics**											
Non-use	190/471	1.00	1.00		213/471	1.00	1.00		13/471	1.00	1.00
Use	52/117	0.84 (0.60; 1.17)	1.03 (0.68; 1.56)		66/117	0.90 (0.67; 1.21)	0.97 (0.67; 1.41)		7/117	1.03 (0.39; 2.74)	0.57 (0.16; 1.98)
low^b^	28/58	0.88 (0.57; 1.34)	0.99 (0.61; 1.62)		34/58	0.89 (0.60; 1.31)	0.90 (0.58; 1.41)				
high	24/59	0.80 (0.52; 1.24)	1.09 (0.64; 1.87)		32/59	0.91 (0.61; 1.33)	1.07 (0.67; 1.73)				
**Beta blockers**											
Non-use	165/422	1.00	1.00		187/422	1.00	1.00		11/422	1.00	1.00
Use	77/166	1.10 (0.83; 1.46)	1.43 (0.98; 2.08)		92/166	1.11 (0.85; 1.44)	1.32 (0.94; 1.86)		9/166	1.34 (0.54; 3.33)	0.89 (0.29; 2.73)
low^b^	44/82	1.37 (0.97; 1.93)	**1.67 (1.10; 2.53)**		50/82	1.31 (0.95; 1.81)	1.50 (1.02; 2.21)				
high	33/84	0.87 (0.60; 1.28)	1.12 (0.69; 1.83)		42/84	0.94 (0.67; 1.32)	1.10 (0.71; 1.71)				
**Calcium channel blockers**											
Non-use	216/515	1.00	1.00		240/515	1.00	1.00		13/515	1.00	1.00
Use	26/73	0.66 (0.43; 1.00)	0.75 (0.45; 1.23)		39/73	0.85 (0.60; 1.23)	0.95 (0.62; 1.45)		7/73	2.21 (0.83; 5.88)	1.75 (0.53; 5.72)
low^b^	10/34	0.57 (0.30; 1.08)	0.84 (0.41; 1.73)		16/34	0.80 (0.48; 1.33)	1.05 (0.58; 1.90)				
high	16/39	0.73 (0.43; 1.24)	0.70 (0.39; 1.26)		23/39	0.90 (0.58; 1.41)	0.89 (0.54; 1.48)				
**Angiotensin receptor blockers**											
Non-use	218/526	1.00	1.00		245/526	1.00	1.00		13/526	1.00	1.00
Use	24/62	0.81 (0.52; 1.24)	0.73 (0.43; 1.23)		34/62	0.99 (0.68; 1.43)	0.89 (0.57; 1.39)		7/62	**3.07 (1.17; 8.06)**	2.45 (0.72; 8.31)
low^b^	12/31	0.87 (0.48; 1.57)	0.64 (0.31; 1.30)		18/31	1.12 (0.69; 1.83)	0.90 (0.50; 1.62)				
high	12/31	0.75 (0.42; 1.36)	0.82 (0.43; 1.57)		16/31	0.87 (0.52; 1.45)	0.88 (0.49; 1.56)				
**ACE inhibitors**											
Non-use	187/443	1.00	1.00		212/443	1.00	1.00		14/443	1.00	1.00
Use	55/145	**0.68 (0.50; 0.94)**	**0.55 (0.36; 0.83)**		67/145	**0.70 (0.52; 0.93)**	**0.55 (0.38; 0.80)**		6/145	0.67 (0.25; 1.81)	0.56 (0.17; 1.84)
low^b^	27/72	0.69 (0.45; 1.05)	0.65 (0.40; 1.05)		33/72	**0.66 (0.46; 0.94)**	**0.63 (0.41; 0.97)**				
high	28/73	0.68 (0.45; 1.02)	**0.46 (0.28; 0.77)** ^*^		34/73	0.76 (0.51; 1.13)^*^	**0.48 (0.30; 0.76)** ^*^				

^a^Cox model, adjusted for age, place of residence, CCI, stage, histology, surgery, chemotherapy, radiotherapy, use of anticoagulants, statins, antidiabetics, antihypertensive medications 1-year pre-diagnosis. Mutually adjusted for the use of antihypertensive medications 1-year after diagnosis

^b^low usage: ≤median of DDD amount; high usage: >median of DDD amount. No results by amount categories were included in the non-cancer mortality analysis due to insufficient numbers of patients to allow meaningful analysis

^*^ *p* for trend < 0.05

In lag-time analysis, no association between the use of SNS-AH, diuretics, BBs, CCBs or ARBs and ovarian cancer-specific mortality was observed (Table 3), although the risk decrease for ARB users became more pronounced after excluding first two years after diagnosis, HR 0.55, 95% CI: 0.29–1.04. The association with ACE inhibitors’ use stayed decreased, after excluding first two years after diagnosis HR 0.58, 95% CI: 0.37–0.93.

**Table 3:** Antihypertensive medications use and risk of ovarian cancer mortality: lag time analyses

Antihypertensive medications use		Follow-up excluding first 2 years after diagnosis	
	HR (95% CI)^a^	Deaths/ Cases, n	HR (95% CI)^a^
**SNS-AH**			
Non-use	1.00	156/467	1.00
Use	1.05 (0.61; 1.80)	18/43	1.10 (0.58; 2.10)
**Diuretics**			
Non-use	1.00	134/410	1.00
Use	1.03 (0.68; 1.56)	40/100	1.01 (0.62; 1.65)
**Beta blockers**			
Non-use	1.00	116/366	1.00
Use	1.43 (0.98; 2.08)	58/144	1.47 (0.94; 2.29)
**Calcium channel blockers**			
Non-use	1.00	153/446	1.00
Use	0.75 (0.45; 1.23)	21/64	0.78 (0.44; 1.40)
**Angiotensin receptor blockers**			
Non-use	1.00	160/461	1.00
Use	0.73 (0.43; 1.23)	14/49	0.55 (0.29; 1.04)
**ACE inhibitors**			
Non-use	1.00	131/380	1.00
Use	**0.55 (0.36; 0.83)**	43/130	**0.58 (0.37; 0.93)**

^a^Cox model, adjusted for age, place of residence, CCI, stage, histology, surgery, chemotherapy, radiotherapy, use of anticoagulants, statins, antidiabetics, antihypertensive medications 1-year prior to diagnosis. Mutually adjusted for the use of antihypertensive medications 1-year after diagnosis

We next examined the effect of treatment within 1-year post-diagnosis period with ARBs and ACE inhibitors separately and combined. We found, that users of either ARBs or ACE inhibitors separately had lower cancer-specific death risk (HR 0.84, 95% CI: 0.47–1.51 and HR 0.59, 95% CI: 0.38–0.92, respectively) (Supplementary Table 1, Additional file 1). Consumption of both ARBs and ACE inhibitors during 1 year after diagnosis period showed markedly increased beneficial effect (HR 0.26, 95% CI: 0.08–0.89). When analyses were restricted to women > 50 years old at diagnosis, inverse association between ACE inhibitor use and ovarian cancer mortality remained, whereas the risk estimates for associations between BBs or CCBs use and ovarian cancer mortality were attenuated (Supplementary Table 2, Additional file 1).

### Stratified analysis by patients’ characteristics

A subgroup analysis of post-diagnosis use of SNS-AH and Diuretics did not show associations with the risk of ovarian cancer-specific mortality (Table 4).

**Table 4:**
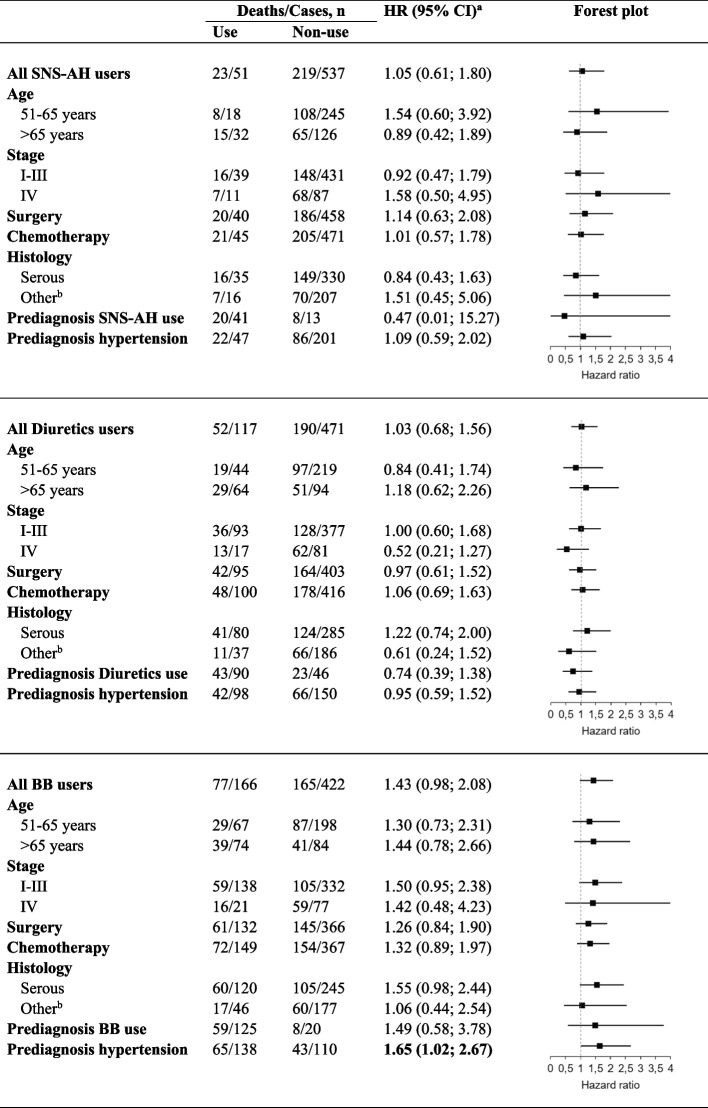
Subgroup analyses of the association between Centrally and peripherally acting antiadrenergic agents (SNS-AH), Diuretics or Beta-blockers (BBs) use and the ovarian cancer mortality risk

^a^Cox model, adjusted for age, place of residence, CCI, stage, histology, surgery, chemotherapy, radiotherapy, use of anticoagulants, statins, antidiabetics, antihypertensive medications 1-year pre-diagnosis. Mutually adjusted for the AH medications use 1-year after diagnosis. bIncluding mucinous, endometrioid and other

In the stratified analyses evaluating post-diagnosis use of BBs, women with pre-diagnosis hypertension who took BBs were more likely to die from ovarian cancer (HR 1.65, 95% CI: 1.02–2.67) (Table 4). Associations with BBs use by other subgroups (age, stage, anticancer treatment) generally were similar, although there was a suggestion that BBs use was associated with an increased ovarian cancer death risk in women with serous cancers (HR 1.55, 95% CI: 0.98–2.44), but not in those with non-serous cancers (HR 1.06, 95% CI: 0.44–2.54). In stratified analysis ovarian cancer patients users of CCBs tended to have somewhat lower risk of mortality compared to non-users; however, the results by age and histology were inconsistent. A statistically significantly decreased cancer-specific mortality was observed in subgroups of age > 65 years (HR 0.47, 95% CI: 0.23–0.95) and serous histology (HR 0.49, 95% CI: 0.27–0.90), but not among those 51–65 years old or with “Other” histology (Table 5).

**Table 5:**
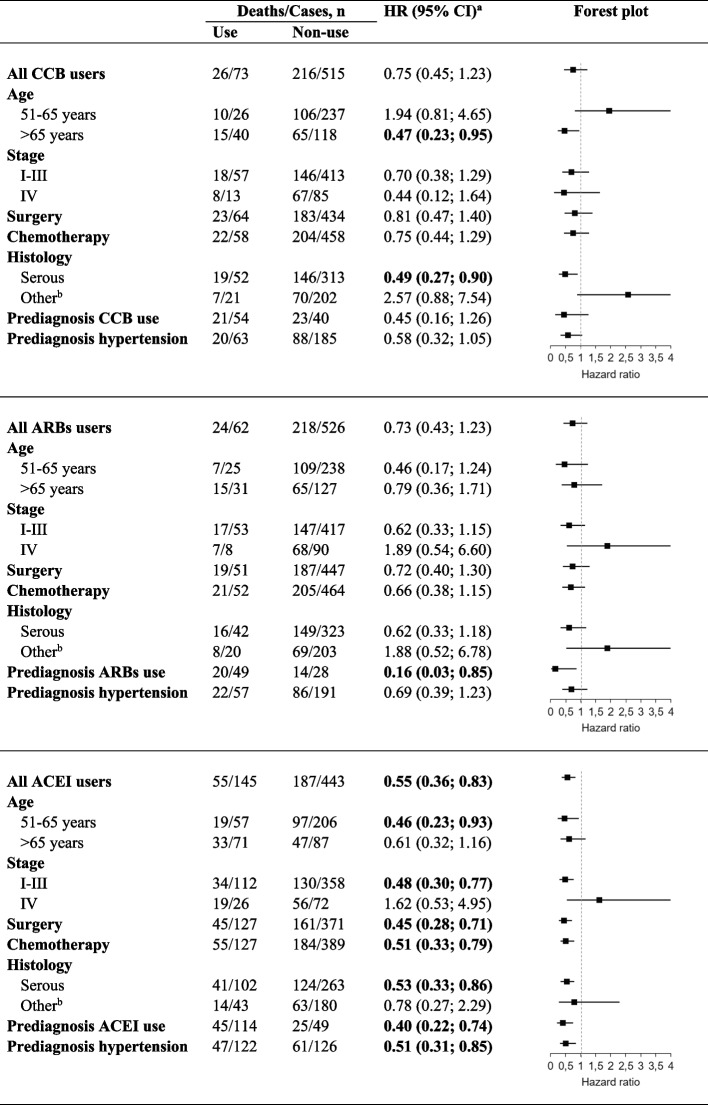
Subgroup analyses of the association between Calcium-channel blockers (CCBs), Angiotensin-receptor blockers (ARBs) or Angiotensin-converting enzyme inhibitors (ACEIs) use and risk of ovarian cancer mortality

^a^Cox model, adjusted for age, place of residence, CCI, stage, histology, surgery, chemotherapy, radiotherapy, use of anticoagulants, statins, antidiabetics, antihypertensive medications 1-year pre-diagnosis. Mutually adjusted for the AH medications use 1-year after diagnosis. bIncluding mucinous, endometrioid and other

A protective association between ARBs and ovarian cancer-specific mortality was observed in pre-diagnosis ARBs users (HR 0.16, 95% CI: 0.03–0.85). Not statistically significantly reduced risk in ARBs users was observed in subgroups of stage I–III (HR 0.62, 95% CI: 0.33–1.15), chemotherapy treatment (HR 0.66, 95% CI: 0.38–1.15) and serous adenocarcinoma (HR 0.62, 95% CI: 0.33–1.18). Mortality was markedly reduced among patients aged 51–65 years who had stage I-III ovarian cancer (HR 0.16, 95% CI: 0.04–0.67, data not shown).

The analyses confirmed previous results showing that users of ACE inhibitors had a statistically significantly decreased risk of ovarian cancer-specific mortality. There were mortality reductions in subgroups of age 51–65 years (HR 0.46, 95% CI: 0.23–0.93), stage I–III (HR 0.48, 95% CI: 0.30–0.77), surgery (HR 0.48, 95% CI: 0.28–0.71), chemotherapy (HR 0.51, 95% CI: 0.33–0.79), pre-diagnosis ACE inhibitors users (HR 0.40, 95% CI: 0.22–0.74) and patients who had hypertension prior to the ovarian cancer diagnosis (HR 0.51, 95% CI: 0.31–0.85) (Table 5). Furthermore, the mortality reduction was also observed in subgroup of serous adenocarcinoma (HR 0.53, 95% CI: 0.33–0.86), but not those with non-serous histology (HR 0.78, 95% CI: 0.27–2.29). The association became stronger among ACE inhibitors users aged 51–65 years with stage I-III ovarian cancer (HR 0.26, 95% CI: 0.09–0.76, data not shown).

## Discussion

We evaluated ovarian cancer-specific and all-cause mortality by post-diagnostic AH medication use in a cohort study of Lithuanian women ovarian cancer patients. This study demonstrated an association between ACE inhibitors and around 45% reduced risk of ovarian cancer mortality. The risk of death due to ovarian cancer was related to the amount of ACE inhibitors consumed during the first year after diagnosis and the effect was stronger in patients, having used ACE inhibitors prior to diagnosis. A decreased risk among ACE inhibitors users was observed also in subgroups of patients aged 51–65 years, having serous adenocarcinoma and stage I–III at diagnosis. The association persisted in lag-time analysis, suggesting that the immortal time bias or reverse causality are unlikely explanations.

Our result that ACE inhibitors use after ovarian cancer diagnosis is associated with reduced cancer-specific mortality risk is in agreement with results of previous studies [6–8]. Huang et al. (2021), observed a reduced mortality for post-diagnostic therapy involving ACE inhibitors (HR 0.53, 95% CI: 0.31– 0.91), but no association for post-diagnostic use of other antihypertensive medication [7]. Similarly, Santala et al. reported, that only ACE inhibitors are associated with improved long term ovarian cancer survival when the effect of cardiovascular mortality is taken into account (HR 0.73 95% CI: 0.58–0.91) [6]. In a study by Harding et al., ovarian cancer-specific mortality was found to be lower among women who used ACE inhibitors (HR 0.76, 95% CI: 0.63–0.92) [8].

Our results imply that ovarian cancer patients using ARBs may have somewhat lower cancer-specific mortality risk. This is consistent with a study by Cho et al., which reported association of ARBs use with lower recurrence of epithelial ovarian cancer during primary treatment, and no beneficial impact of CCBs, BBs, and TDs [9]. However, a study among Finnish ovarian cancer patients did not support a reduced risk of ovarian cancer death among post-diagnosis ARBs users [6]. We found that the use of both ACE inhibitors and ARBs during first year after diagnosis had a significantly greater benefit on survival compared to each medication class alone, although the result was based on few outcomes. The possible mechanisms underlying the observed reduction of cancer-specific death risk following the ARB and ACE inhibitors medication use include reduction of inflammation, tumour angiogenesis, invasion and peritoneal dissemination in human ovarian cancer cells by blockage of angiotensin receptors (both Angiotensin II type 1 receptor (AT1R) and Angiotensin II type 2 receptor (AT2R)) [6, 12]. According to Suganuma et al., AT1R expression has been observed in 85% of invasive ovarian carcinomas, whereas it was not detected on surface epithelium of the normal ovary [23]. Authors also detected angiotensin converting enzyme (ACE) in ovarian tumor stroma. These findings are consistent with observation of synergistic anti-tumour effect of treatment with ARB and AT2R agonist medication combined in comparison to either alone and suggest a therapeutic benefit in the dual regulation of AT1R and AT2R in ovarian cancer [24].

Our nationwide population-based cohort study showed that the SNS-AH use was not associated with survival outcomes in ovarian cancer patients. The use of SNS-AH is relatively high in Lithuania − 31.5 DDD/TID in Lithuania in 2012 compared to less than 5 DDD/TID in EE and Scandinavian countries [20]. This enabled us to analyse the relationship between SNS-AH use and ovarian cancer mortality. This has, to our knowledge, not been previously examined and therefore makes our study unique among all other studies examining the impact of the use of AH medications among patients with ovarian cancer.

We found, that post-diagnostic use of diuretics was not related to cancer-specific mortality, in agreement with most previous investigations [6–9]. In our study, users of CCBs had somewhat decreased ovarian cancer mortality; however, the HRs among all study subjects and in subgroup analyses were generally not statistically significant, also inconsistent. Thus, the possibility of chance findings, as well as residual confounding cannot be excluded in the observed results. In agreement with our study, no association with post-diagnostic use of CCBs was found in several previous studies [6–9].

The present cohort study suggests a tendency towards poorer survival among post-diagnostic BBs users. The association remained similar in stratified analysis, but not in sensitivity (restricted to > 50 years old women) or dose–response analysis. Studies analysing the association between the use of BBs and survival among ovarian cancer patients report contradictory results. In most studies BBs users showed no association [6, 7, 9, 12, 13] or better survival outcomes [10, 25, 26]. A study by Harding et al. (2018) found ovarian cancer-specific mortality to be lower among women who used nonselective BBs, but not in selective BBs users [8]. Our findings largely agree with the results by Gonzalez et al. (2020), Wen et al. (2021) and Couttenier et al. (2019) studies that reported an association between the use of BBs and increased mortality or worse prognosis in patients with ovarian cancer [12–14]. Notably, in Gonzalez et al. (2020) study the majority of patients took cardioselective β-blockers [12]. The different results for BBs and cancer mortality in various studies are most likely due to the different mechanisms of action of BBs. Non-selective BBs block both β1 adrenergic and β2 adrenergic receptors, and it has been suggested that they are associated with lower ovarian cancer mortality [8, 10, 11, 26, 27]. In contrast, for selective BBs, which specifically activate cardiac β1 adrenergic receptors, association with higher ovarian cancer mortality or no relationship was shown [8, 11, 13, 14]. Pharmacological studies of ovarian and other cancer (breast, prostate) models have also reported importance of β2- or β3-adrenergic receptors and an inhibiting effect on tumor progression of non-selective β-antagonists, whereas more common β1 antagonists (i.e., atenolol) had no protective effect [28]. Notably, selective BBs use is a standard practice for arterial hypertension treatment in Lithuania [29]. As all BBs users were β1 antagonist users in our study population, this might explain no protective effect observed in BBs users.

The ovarian cancer-related mortality hazard rate patterns differed somewhat between “Serous adenocarcinoma” and “Other” histological type categories for some of the classes of AH medications. Namely, in women with serous cancers CCB and ACE inhibitor use was associated with a significantly decreased ovarian cancer death risk, but no such association was observed in “Other” histological type category. These results may be due to the small numbers of deaths and data variability, e.g., the non-serous ovarian cancer in CCBs user groups consisted of only 7 cases.

The strength of our study is a nationwide study cohort, covering all ovarian cancer patients in Lithuania diagnosed during 2013–2015. We used the NHIF data on time and amount of medication purchased that was detailed and free of recall bias. This allowed us to analyse drugs use by each antihypertensive drugs class separately, taking into account patients’ simultaneous use of multiple antihypertensive drugs within the year since diagnosis and prior to their diagnosis as well as use of other drugs (statin, antidiabetic and antithrombotic medicines). A 1-year exposure assessment period prior to the start of follow-up time was introduced in order to reduce the effect of immortal time bias. The available information on clinical factors including cancer stage, histology, cancer therapies, hypertension and other comorbidities, allowed subgroup analyses and adjustments for potential confounders.

The main limitation of the study is relatively low number of ovarian cancer deaths in this cohort of ovarian cancer patients and therefore limited statistical power in subgroups analyses. Thus, we cannot entirely rule out the possibility of chance findings. Particularly, the sample size was reduced in stratified analyses, thus the error may have occurred. Our data on antihypertensive medication use was based on recorded medication purchases, and we had no information whether the drugs were actually consumed. Treciokiene et al. (2022) have found that 57% of patients were non-persistent to antihypertensive therapy at the end of the first treatment year in Lithuanian population [30]. As our study population is older and consists of cancer patients, it is likely that the non-adherence rates are lower compared to general population. Nevertheless, there is a potential for misclassification of exposure because some of non-users might have been incorrectly classified as users. Because of the study design (cohort study, data source – NHIF database), misclassification of AH medication consumption would be non-differential and would tend to attenuate the effect estimates towards null. In addition, the subgroup analysis among pre-diagnosis users of AH medications would have reduced this misclassification, as people generally do not refill prescriptions if they are not using medication.

Antihypertensive medication users differed from non-users on some characteristics such as age at diagnosis or comorbidities; however, inclusion of these variables in the multivariate models as well as stratified analyses should have efficiently controlled for the differences. Further, we had no information on lifestyle factors such as smoking, BMI, diet or physical activity, and they may have an influence on ovarian cancer survival [31]. We cannot rule out residual confounding by factors that we have not accounted for; however, the association found with ACE inhibitor use was strong and residual confounding probably cannot explain this association entirely. Furthermore, our result of an association between the post-diagnostic ACE inhibitor use and lower ovarian cancer mortality is consistent with previously reported data, adjusted for BMI, smoking and other potentially confounding variables [7]. We also performed a sensitivity analysis restricted to study participants above 50 years old. The risk estimates for ACE inhibitor use stayed decreased; however, HRs were attenuated for BBs and CCBs use. This indicates, that the possibility of residual confounding by factors not addressed cannot be completely ruled out and the results should be interpreted with caution.

## Conclusions

We found, that using ACE inhibitors following the ovarian cancer diagnosis is associated with improved cancer-specific survival in ovarian cancer patients. The results might be affected by systematic differences between the users and non-users; however, the observed reduction in risk in users of ACE inhibitors compared to non-users is consistent with previous findings; moreover, the risk decrease in dose-response, stratified and lag-time analysis support causal association. Our findings imply that treatment of cardiovascular conditions with ACE inhibitors may have an impact on survival in ovarian cancer patients; however, further studies in larger cohorts and randomised trials are needed to confirm this result.

### Supplementary Information

**Supplementary Material 1.**

## Data Availability

The data that support the findings of this study are available from the Lithuanian Cancer Registry and the National Health Insurance Fund but restrictions apply to the availability of these data, which were used under license for the current study, and so are not publicly available. Data are however available from the corresponding author upon reasonable request and with permission of Vilnius Regional Biomedical Research Ethics Committee.
