# Supplementary material for: Evaluation of antihypertensive medications use and survival in patients with ovarian cancer: a population-based retrospective cohort study
Source: BMC Womens Health. 2024 Mar 4;24:155. doi: 10.1186/s12905-024-02983-7 (PMC10913626; doi:10.1186/s12905-024-02983-7)
Supplement: Supplementary file 1 — Supplementary Material 1. [file 12905_2024_2983_MOESM1_ESM.docx]

| **Supplementary Table 1.** Angiotensin receptor blockers (ARBs) and ACE inhibitors (ACEIs) use and risk of ovarian cancer and all-cause mortality. | | | | |
| --- | --- | --- | --- | --- |
| **ARBs/ACE inhibitors use** | **Ovarian cancer mortality** | | **All-cause mortality** | |
|  | **Deaths/ Cases, n** | **HR (95% CI)^a^** | **Deaths/ Cases, n** | **HR (95% CI)^b^** |
| Non-use | 166/397 | 1.00 | 183/397 | 1.00 |
| ARBs, no ACEIs | 21/46 | 0.84 (0.47; 1.51) | 29/46 | 0.99 (0.60; 1.65) |
| ACEIs, no ARBs | 52/129 | **0.59 (0.38; 0.92)** | 62/129 | **0.59 (0.40; 0.88)** |
| ARBs and ACEIs | 3/16 | **0.26 (0.08; 0.89)** | 5/16 | **0.38 (0.14; 0.99)** |
| ^a^Fine-Gray model, adjusted for: age, place of residence, CCI, stage, histology, surgery, chemotherapy, radiotherapy, use of anticoagulants, statins, antidiabetics, use of antihypertensive medications 1-year prior to diagnosis and use of antihypertensive medications one year after diagnosis.  ^b^Cox model, adjusted for: age, place of residence, CCI, stage, histology, surgery, chemotherapy, radiotherapy, use of anticoagulants, statins, antidiabetics, use of antihypertensive medications 1-year prior to diagnosis and use of antihypertensive medications one year after diagnosis. | | | | |

| **Supplementary Table 2.** Antihypertensive (AH) medications use and risk of ovarian cancer mortality. Analyses restricted to women more than 50 years old at diagnosis. | | | | |
| --- | --- | --- | --- | --- |
| **AH medication use** | **Ovarian cancer mortality** | | | |
|  | **Deaths/ Cases, n** | **HR (95% CI)^a^** | | |
|  |  | **All women** | **Age >50 years^a^** | |
| **SNS-AH** | |  |  | |
| Non-use | 219/537 | 1.00 | 1.00 | |
| Use | 23/51 | 1.05 (0.61; 1.80) | 1.15 (0.66; 1.98) | |
| low^b^ | 10/25 | 1.01 (0.48; 2.10) | 1.11 (0.53; 2.33) | |
| high | 13/26 | 1.08 (0.56; 2.11) | 1.18 (0.60; 2.30) | |
| **Diuretics** |  |  |  | |
| Non-use | 190/471 | 1.00 | 1.00 | |
| Use | 52/117 | 1.03 (0.68; 1.56) | 0.92 (0.60; 1.43) | |
| low^b^ | 28/58 | 0.99 (0.61; 1.62) | 0.80 (0.48; 1.36) | |
| high | 24/59 | 1.09 (0.64; 1.87) | 1.11 (0.63; 1.92) | |
| **Beta blockers** | | | |  |
| Non-use | 165/422 | 1.00 | 1.00 | |
| Use | 77/166 | 1.43 (0.98; 2.08) | 1.31 (0.88; 1.95) | |
| low^b^ | 44/82 | **1.67 (1.10; 2.53)** | 1.48 (0.94; 2.33) | |
| high | 33/84 | 1.12 (0.69; 1.83) | 1.12 (0.68; 1.85) | |
| **Calcium channel blockers** | | | |  |
| Non-use | 216/515 | 1.00 | 1.00 | |
| Use | 26/73 | 0.75 (0.45; 1.23) | 0.81 (0.49; 1.35) | |
| low^b^ | 10/34 | 0.84 (0.41; 1.73) | 0.87 (0.40; 1.86) | |
| high | 16/39 | 0.70 (0.39; 1.26) | 0.78 (0.43; 1.42) | |
| **Angiotensin receptor blockers** | | | |  |
| Non-use | 218/526 | 1.00 | 1.00 | |
| Use | 24/62 | 0.73 (0.43; 1.23) | 0.74 (0.42; 1.28) | |
| low^b^ | 12/31 | 0.64 (0.31; 1.30) | 0.62 (0.28; 1.35) | |
| high | 12/31 | 0.82 (0.43; 1.57) | 0.84 (0.43; 1.64) | |
| **ACE inhibitors** | | | |  |
| Non-use | 187/443 | 1.00 | 1.00 | |
| Use | 55/145 | **0.55 (0.36; 0.83)** | **0.58 (0.38; 0.90)** | |
| low^b^ | 27/72 | 0.65 (0.40; 1.05) | 0.65 (0.39; 1.08) | |
| high | 28/73 | **0.46 (0.28; 0.77) ^*^** | **0.52 (0.31; 0.88)^*^** | |
| ^a^Cox model, adjusted for age, place of residence, CCI, stage, histology, surgery, chemotherapy, radiotherapy, use of anticoagulants, statins, antidiabetics, antihypertensive medications 1-year pre-diagnosis. Mutually adjusted for the use of antihypertensive medications 1-year after diagnosis.  ^b^low usage: ≤median of DDD amount; high usage: >median of DDD amount.  ^*^p for trend <0.05. | | | | |
